# Trichlorfon and spinosad resistance survey and preliminary determination of the resistance mechanism in Pakistani field strains of ***Bactrocera dorsalis***

**DOI:** 10.1038/s41598-018-29622-0

**Published:** 2018-07-25

**Authors:** Hafiz Azhar Ali Khan, Waseem Akram

**Affiliations:** 1 Institute of Agricultural Sciences, University of the Punjab, Lahore, Pakistan; 2 Department of Entomology, University of Agriculture, Faisalabad, Pakistan

## Abstract

The use of insecticides has been a primary tool to manage *Bactrocera dorsalis* in Pakistan; however, recent reports of field control failures necessitate mapping out the insecticide resistance problem. Therefore, eight field strains from Pakistan, were evaluated for their resistance against trichlorfon and spinosad. Compared with a reference strain, six field strains showed high levels of resistance to trichlorfon, while two field strains expressed intermediate resistance. In case of spinosad, five field strains fell in the susceptible range, whereas, the rest of the strains represented minor resistance. Correlation analysis between LD_50_ values of trichlorfon and spinosad of all the field strains revealed non-significant association, suggesting the possibility of lack of cross-resistance between both insecticides. Synergism bioassays implementing *S*,*S*,*S*-tributylphosphorotrithioate (DEF) and piperonyl butoxide (PBO) revealed that the LD_50_ values of trichlorfon in the presence of either DEF or PBO in seven field strains were significantly reduced. However, DEF and PBO had a non-significant effect on synergizing spinosad toxicity. The results revealed resistance to trichlorfon in field strains of *B*. *dorsalis*, which might be metabolic-based. Absence or minor resistance to spinosad and lack of cross-resistance to trichlorfon, suggest that spinosad could be a potential candidate for managing *B*. *dorsalis*.

## Introduction

*Bactrocera dorsalis* (Hendel, 1912) is one of the most damaging pests of fruits throughout the Pacific and South-East Asia^1^, including Pakistan^2^. This pest has also been considered an important quarantine pest in different parts of the world. About 250 host plants, including citrus, peach, carambola, mandarin, mango, guava, chili pepper and coffee, have been reported to be attacked by *B*. *dorsalis*^3,4^. Internal feeding habits by the larvae of *B*. *dorsalis* and decaying of fruits at points where adult females insert their ovipositor make the fruits unacceptable by the consumers which ultimately cause heavy financial losses.

The use of insecticides has been considered as a primary tool for the successful control of *B*. *dorsalis*^1^; however, the benefits of this tool are usually compromised due to the development of insecticide resistance. A number of cases concerning the development of insecticide resistance in *B*. *dorsalis* have been reported from different parts of the world^1,4–7^; however, to the best of authors’ knowledge, there is no reported case of resistance in *B*. *dorsalis* from Punjab, Pakistan. Since, insecticide resistance is a spatio-temporal phenomenon^8^, it is necessary to know the resistance situation in Pakistan for the better management of *B*. *dorsalis*. Previously, insecticide resistance in *B*. *zonata* has been reported to different organophosphate and pyrethroid insecticides from some parts of the Punjab province, Pakistan^2,9,10^. In Pakistan, organophosphates, particularly trichlorfon, are more frequently used for the management of tephritid fruit flies^2^; however, recently farming communities have reported field control failures by the use of trichlorfon (personal communication with farmers and agricultural extension workers). In response to this, spinosad, a bacterial insecticide derived from *Saccharopolyspora spinosa* Mertz & Yao, has recently been included as an alternate to trichlorfon for the management of tephritid fruit flies in Punjab, Pakistan.

Assessment of insecticide resistance is very important to devise an effective management strategy against insect pests^11^. Detection of insecticide resistance in *B*. *dorsalis* can help to adopt alternate measures to slow the spread of resistance. Therefore, keeping in view the economic importance of *B*. *dorsalis* and field control failures, a study was planned to assess the level of resistance to one of the most commonly used insecticide (trichlorfon) and the newly introduced insecticide (spinosad) for the management of *B*. *dorsalis* in Punjab, Pakistan. The data could be helpful to devise an effective management strategy for *B*. *dorsalis*.

## Materials and Methods

### Insects

Eight field strains of *B*. *dorsalis* collected from different localities and hosts (Table 1) were maintained under the laboratory conditions 25 ± 2 °C and 12 L: 12D photoperiod, to get the homogenous and sufficient number of insects in bioassays. The adults were maintained in mesh cages (40 × 30 × 30 cm) provided with maize-based artificial diet^4^ and fresh fruits for egg laying. A laboratory reference strain (Ref.) was maintained in 2013 from a strain of insects inhabiting untreated guava trees at the University of the Punjab, Lahore. The Ref. strain was maintained in the laboratory as described above.

**Table 1:** Selected localities, collection period and host plants for the collection of *Bactrocera dorsalis*.

Location	Coordinates	Collection period	Host
Rahim Yar Khan (RN)	28.4212° N, 70.2989° E	July 2015	Mango
Bahawalpur (BR)	29.3957° N, 71.6833° E	July 2015	Mango
Kasur (KR)	31.1165° N, 74.4494° E	October 2015	Guava
Lahore (LR)	31.5546° N, 74.3572° E	October 2015	Guava
Multan (MN)	30.1984° N, 71.4687° E	July 2016	Mango
Jhang (JG)	31.2601° N, 72.3193° E	August 2016	Mango
Sargodha (SA)	32.0837° N, 72.6719° E	October 2016	Sweet lime/sweet orange
Faisalabad (FD)	31.4187° N, 73.0791° E	October 2016	Guava

### Insecticides

Technical-grade spinosad, trichlorfon, *S*,*S*,*S*-tributylphosphorotrithioate (DEF) and piperonyl butoxide (PBO) (purity >98%; Chem Service Inc, West Chester PA) were used in bioassays.

### Bioassays

Assays were done following the methodology described by Hsu, *et al*.^1^. Working insecticide solutions, with a range of doses having >0% and <100% mortality, were prepared in acetone. One-microliter of the appropriate solution was applied onto the thoracic tergum of adult flies (3–5-day-old) using a micropipette (0.1–2 µL, Acura ® *manual* 825, Socorex, Switzerland). Flies in the control group received acetone only. The range of doses tested varied for different strains being tested. For spinosad, the range of doses was 0 to 32 ng/fly for the Ref. strain, and 0 to 96 ng/fly for field strains. For trichlorfon, doses used to test ranged from 0 to 48 ng/fly (Ref. strain) and 0 to 2460 ng/fly (field strains). Treated flies were shifted to perforated plastic jars (250 ml) containing a cotton swab (2 cm) moistened with a liquid food [sugar(4 parts):yeast(1 part):water(5 parts)] and kept under said laboratory conditions. For the synergism bioassays, PBO and DEF were applied onto the dorsal thorax of all the strains at the rate of 1 µg per fly for two hours before the insecticidal bioassays. Mortality data were recorded 48 h after the insecticide treatment.

### Data analyses

Mortality scores were analyzed by Probit analysis using SPSS 16, to calculate median lethal dose (LD_50_) values. Resistance ratios (RRs) were calculated by dividing LD_50_ value of a field strain by the corresponding LD_50_ value of the Ref. strain. Jin, *et al*.^12^ criterion was used to classify resistance levels: susceptible (<3-fold RR), minor resistance (3–5-fold RR), low resistance (5–10-fold RR), intermediate resistance (10–40-fold RR), high resistance (40–160-fold RR), and extremely high resistance (RR >160-fold). Significant differences between any two (laboratory Ref. vs field strains) LD_50_ values were determined by calculating the 95% fiducial limit (FL) of the RR values (SR) at the LD_50_ level. If the 95% FL includes 1, the RR values are not significantly different. Synergism ratios were calculated by dividing the LD_50_ value without synergist by the LD_50_ value with synergist (PBO or DEF), and the significance of synergistic effects were assessed by calculating the 95% FL of the synergism ratio (SR) at the LD_50_ level^13^, as stated above.

### Ethical statement

The article deals with *B*. *dorsalis* which is an invertebrate. The study was conducted according to the standard guidelines and regulations. The study/bioassay protocol was approved by the research projects evaluation committee of the Institute of Agricultural Sciences, University of the Punjab, Lahore.

## Results

### Baselines susceptibility to spinosad and trichlorfon

The laboratory reared reference strain (Ref.) of *B*. *dorsalis* showed relatively much higher susceptibilities to trichlorfon and spinosad as compared to field strains (based on 95% FLs of RRs didn’t include 1). The LD_50_ values of trichlorfon and spinosad were 8.42 [FL = 7.19–9.85] and 5.39 [FL = 4.5–6.4] ng/fly, respectively (Table 2). These values were used as baselines for evaluating resistance in different field strains.

**Table 2:** Toxicity of trichlorfon and spinosad in different strains of *Bactrocera dorsalis*.

Insecticide	Strain	n^*^	LD_50_^**^ (95% FL^***^)	Slope (SE)	χ^2^	df	*p*	RR^****^ (95% FL)
Trichlorfon	Ref.	420	8.42 (7.19–9.85)	2.45 (0.21)	5.83	4	0.21	1.0
	RN	360	471.48 (392.37–561.04)	2.19 (0.22)	5.26	3	0.15	55.99 (44.11–71.11)^+^
	BR	360	509.56 (430.30–599.19)	2.41 (0.24)	0.98	3	0.81	60.52 (48.13–76.12)^+^
	MN	360	738.01 (619.79–885.69)	2.18 (0.22)	3.23	3	0.36	87.65 (69.06–111.29)^+^
	JG	360	568.93 (483.50–672.67)	2.41 (0.23)	1.44	3	0.70	67.57 (53.75–84.97)^+^
	LR	420	188.23 (158.92–221.17)	2.35 (0.21)	3.19	4	0.53	22.36 (17.77–28.13)^+^
	SA	360	447.30 (380.12–522.56)	2.58 (0.25)	1.71	3	0.64	53.12 (42.44–66.52)^+^
	KR	360	503.84 (456.26–596.07)	2.34 (0.23)	3.15	3	0.37	59.84 (47.52–75.39)^+^
	FD	420	172.50 (141.77–206.82)	1.98 (0.18)	2.03	4	0.73	20.49 (16.01–26.23)^+^
Spinosad	Ref.	420	5.39 (4.5–6.4)	2.02 (0.17)	5.20	4	0.27	1.0
	RN	420	14.32 (12.20–16.86)	2.37 (0.20)	6.28	4	0.18	2.66 (2.09–3.39)^+^
	BR	420	14.10 (12.02–16.59)	2.38 (0.20)	4.91	4	0.30	2.62 (2.05–3.33)^+^
	MN	420	16.95 (14.48–19.97)	2.43 (0.21)	0.60	4	0.96	3.14 (2.47–4.01)^+^
	JG	420	11.64 (9.63–14.08)	1.86 (0.16)	4.46	4	0.35	2.16 (1.66–2.81)^+^
	LR	420	14.17 (12.14–16.56)	2.52 (0.21)	5.57	4	0.23	2.63 (2.07–3.34)^+^
	SA	420	14.49 (12.19–17.17)	2.15 (0.19)	3.65	4	0.46	2.69 (2.10–3.45)^+^
	KR	420	17.88 (15.02–20.52)	2.67 (0.22)	8.27	4	0.08	3.32 (2.62–4.20)^+^
	FD	420	25.47 (21.49–30.47)	2.17 (0.19)	4.14	4	0.39	4.73 (3.68–6.07)^+^

*n = number of insects used in bioassays, **LD_50_ = median lethal dose (ng/fly), ***FL = fiducial limits, ****RR = resistance ratio, calculated by dividing LD_50_ value of a field strain by the corresponding LD_50_ value of the Ref. strain, and +  = significantly different from the Ref. strain based on 95% FLs of RRs didn’t include 1. The same applies to the Table 3.

### Response of field strains

In total, eight field strains collected form eight localities were evaluated for resistance to trichlorfon and spinosad. Of these, six strains (RN, BR, MN, JG, SA, and KR) showed high levels of resistance to trichlorfon (LD_50s_ = 471.48–738.01 ng/fly; RR = 53.12–87.65-fold), while two strains (LR and FD) showed intermediate resistance levels to trichlorfon (LD_50s_ = 172.50–188.23 ng/fly; RR = 20.49–22.36-fold). Among field strains, the FD and LR strains were the most susceptible to trichlorfon while the MN strain was the most resistant strain. In case of spinosad, five of field strains (RN, BR, JG, LR, and SA) fell in the susceptible range (LD_50s_ = 11.64–14.49 ng/fly; RR = 2.16–2.69-fold), whereas, the rest of the strains (MN, KR, and FD) represented minor resistance (LD_50s_ = 16.95–25.47 ng/fly; RR = 3.14–4.73-fold) (Table 2). Spinosad showed the highest toxicity to the JG, BR, LR, RN and SA strains (all were at par based on overlapping 95% FLs) followed by the MN and KR strains, while the FD strain showed the least susceptibility to spinosad.

Correlation analysis between LD_50_ values of trichlorfon and spinosad of all the field strains revealed non-significant association (r = −0.43; p = 0.28), suggesting the possibility of lack of cross-resistance between both insecticides.

### Synergism experiment

The results of synergism bioassays implementing DEF and PBO are shown in the Table 3. The results revealed that the LD_50_ values of trichlorfon in the presence of either DEF or PBO in all the field strains, except the SA strain, were significantly reduced (based on 95% FLs of SRs didn’t include 1) when compared with the LD_50_ values of their respective field strains without any synergist (Table 3). However, DEF and PBO had a non-significant effect on synergizing spinosad toxicity in all the strains of *B*. *dorsalis* (data not shown here).

**Table 3:** Effect of enzyme inhibitors on toxicity of trichlorfon in laboratory and field strains of *Bactrocera dorsalis*.

Strain	Synergist	n	LD_50_ (95% FL)	Slope (SE)	χ^2^	df	*p*	SR* (95% FL)
Ref.	PBO	420	8.44 (7.07–10.13)	2.05 (0.17)	2.00	4	0.73	1.00 (0.79–1.28)^ns^
	DEF	420	10.44 (8.90–12.32)	2.38 (0.21)	4.48	4	0.34	0.81 (0.64–1.01)^ns^
RN	PBO	360	320.41 (267.57–378.21)	2.37 (0.24)	3.98	3	0.26	1.47 (1.15–1.89)^+^
	DEF	360	300.94 (253.88–351.96)	2.60 (0.26)	2.76	3	0.43	1.56 (1.23–1.99)^+^
BR	PBO	420	290.97 (248.06–341.68)	2.38 (0.20)	0.92	4	0.92	1.75 (1.39–2.21)^+^
	DEF	420	240.41 (203.47–283.47)	2.27 (0.19)	4.21	4	0.38	2.12 (1.68–2.68)^+^
MN	PBO	420	384.01 (325.63–455.67)	2.25 (0.20)	3.65	4	0.46	1.92 (1.50–2.46)^+^
	DEF	420	301.20 (260.08–348.83)	2.75 (0.23)	2.54	4	0.64	2.45 (1.94–3.09)^+^
JG	PBO	360	304.82 (259.94–354.93)	2.66 (0.26)	0.88	3	0.83	1.87 (1.49–2.34)^+^
	DEF	360	366.44 (319.82–419.23)	3.27 (0.30)	1.51	3	0.68	1.55 (1.25–1.92)^+^
LR	PBO	360	111.98 (93.30–131.75)	2.50 (0.26)	0.76	3	0.86	1.68 (1.32–2.14)^+^
	DEF	360	96.67 (77.78–116.03)	2.28 (0.25)	1.13	3	0.77	1.94 (1.50–2.52)^+^
SA	PBO	360	502.90 (423.38–596.99)	2.26 (0.23)	1.58	3	0.66	0.88 (0.70–1.12)^ns^
	DEF	360	477.17 (405.73–561.18)	2.45 (0.24)	2.60	3	0.46	0.93 (0.75–1.18)^ns^
KR	PBO	360	371.48 (310.44–440.14)	2.27 (0.23)	2.46	3	0.48	1.35 (1.07–1.73)^+^
	DEF	360	292.88 (243.04–346.37)	2.36 (0.25)	3.23	3	0.36	1.72 (1.35–2.20)^+^
FD	PBO	360	99.79 (80.18–120.03)	2.17 (0.24)	1.83	3	0.61	1.72 (1.31–2.28)^+^
	DEF	360	90.92 (73.55–108.57)	2.34 (0.26)	2.26	3	0.52	1.89 (1.45–2.49)^+^

ns = non-significant.

*Synergism ratio calculated by dividing the LD_50_ of trichlorfon of a locality by the LD_50_ of trichlorfon with synergist of the respective locality.

## Discussion

Synthetic insecticides have been excessively used for the management of *B*. *dorsalis* in Punjab, Pakistan; however, there is no report on the development of insecticide resistance from studied localities. In the present study, eight field strains of *B*. *dorsalis* were evaluated for their resistance against tichlorofon and spinosad. The results have confirmed the development of intermediate to high levels of resistance to trichlorfon, but fortunately the field strains were susceptible to spinosad since all of them exhibited less that 10-fold RRs which cannot warrant their status of being resistant^11,14^.

This study provides first information regarding resistance to trichlorfon and susceptibility status of spinosad in *B*. *dorsalis* from Pakistan. The most probable reason for the high level of resistance to trichlorfon could be due to the fact that the strains were collected from areas with intensive use of trichlorfon for the last many years, since this chemical has been recommended as an important tool for the management of tephritid flies in Punjab, Pakistan^2^. Trichlorfon has also been recommended for the management of tephritid flies in other countries with the reports of resistance development as a consequence of intensive exposures. For instance, a low level of trichlorfon resistance (RR = 10) has been observed in a field strain^15^ and a laboratory strain^1^ of *B*. *dorsalis* from Taiwan. Jin, *et al*.^4^ reported varying levels of trichlorfon resistance in different field strains of *B*. *dorsalis* in mainland China. Of these, one of the field strains showed a high level of resistance with 70.4-fold RR value, while 16 of the field strains exhibited moderate levels of resistance with RR values ranged from 11.5 to 25.8-folds. At least 10 field strains of *B*. *dorsalis* from south China have been reported with moderate levels of resistance to trichlorfon^6,7^. Some field strains of *B*. *cucurbitae* from Hainan Island, China, has also been reported to have minor resistance against trichlorfon^12^. Similarly, moderate to high levels of resistance to tricholrfon in *B*. *zonata* has been reported from some parts of the Punjab province, Pakistan^2,9^.

In the present study, five of the field strains of *B*. *dorsalis* were found susceptible to spinosad while three strains had minor resistance to spinosad. This might be due to the fact that spinosad has recently been included in management programs for tephritid flies. Previously, field strains of *B*. *zonata* from some parts of Punjab have also been reported susceptible to spinosad^2^. However, spinosad resistance in *Bactrocera* spp. has been reported from other countries, and it was linked with a wide spread application of spinosad in baits or cover sprays. For example, *B*. *dorsalis* from Taiwan^5^, *B*. *cucurbitae* from Hawaii & Taiwan^16^ and China^12^, and *B*. *oleae* from the United States^17^, have been reported with varying levels of resistance to spinosad.

The field strains of *B*. *dorsalis* did not show cross-resistance between trichlorfon and spinosad. However, Hsu and Feng^5^ have reported cross-resistance between spinosad and organophosphates (naled and malathion) in *B*. *dorsalis*. There is generally no cross-resistance between spinosad and other insecticides^18–24^ in different insect pests.

Synergism bioassays in the presence of enzyme inhibitors (PBO, DEF) were conducted in all the strains of *B*. *dorsalis* since such bioassays could help to assess the preliminary mechanisms of insecticide resistance^25^. DEF helps to identify if the mechanism of resistance is oxidase- and/or esterase-based, because it inhibits the activities of oxidases and esterases. Similarly, PBO also helps to identify the oxidase-based mechanism of resistance, since it is an inhibitor of mixed function oxidases^26^. The results of the present study revealed that resistance to trichlorfon in all the field strains, except the SA strain, could be due to the activities of oxidases and/or esterase since both enzyme inhibitors synergized the toxicity of trichlorfon. However, there was no effect of either PBO or DEF in synergizing the toxicity of trichlorfon in the susceptible reference strain. Similarly, there was no synergistic effect of both enzyme inhibitors on the toxicity of spinosad in all the strains of *B*. *dorsalis*, suggesting the possibility of resistance mechanisms other than metabolic-based. Previously, Hsu, *et al*.^1^ has also reported the synergistic effect of DEF on the toxicity of trichlorfon in *B*. *dorsalis*. There was generally no synergistic effect of enzyme inhibitors on the toxicity of spinosad^20,27–29^ in other insect pests. However, there is a further need to assess the exact mechanism of resistance in the present strains of *B*. *dorsalis* by doing biochemical analyses and molecular techniques.

Ironically, intermediate to high levels of resistance to trichlorfon were observed in Pakistani strains of *B*. *dorsalis*. This resistance might be the result of over-reliance on trichlorfon in the chemical management plans for *B*. *dorsalis* in Punjab, Pakistan, and is most probably mediated by enhanced activities of detoxifying enzymes. Simultaneously, no or minor resistance to spinosad was observed in the present study with no cross-resistance to trichlorfon. This is encouraging and provides a window to use both insecticides in rotation along with other integrated management practices for *B*. *dorsalis*.
